# Potentiation of imatinib by cilostazol in sensitive and resistant gastrointestinal stromal tumor cell lines involves YAP inhibition

**DOI:** 10.18632/oncotarget.26734

**Published:** 2019-03-05

**Authors:** Pierre Vandenberghe, Marine Delvaux, Perrine Hagué, Christophe Erneux, Jean-Marie Vanderwinden

**Affiliations:** ^1^ Laboratory of Neurophysiology, Faculty of Medicine, Université Libre de Bruxelles, Brussels, Belgium; ^2^ IRIBHM, Faculty of Medicine, Université Libre de Bruxelles, Brussels, Belgium

**Keywords:** KIT, PDE3A, cancer, verteporfin, drug repurposing

## Abstract

Despite the introduction of tyrosine kinase inhibitors, gastrointestinal stromal tumors (GIST) resistance remains a major clinical challenge. We previously identified phosphodiesterase 3A (PDE3A) as a potential therapeutic target expressed in most GIST. The PDE3 inhibitor cilostazol reduced cell viability and synergized with the tyrosine kinase inhibitor imatinib (Gleevec™) in the imatinib-sensitive GIST882 cell line. Here, we found that cilostazol potentiated imatinib also in the imatinib-resistant GIST48 cell line. Cilostazol induced nuclear exclusion, hence inactivation, of the transcriptional co-activator YAP, in a cAMP-independent manner. Verteporfin, a YAP/TEAD interaction inhibitor, reduced by 90% the viability of both GIST882 and GIST48 cells. Our results highlight the potential use of compounds targeting PDE3A or YAP in combined multitherapy to tackle GIST resistance.

## INTRODUCTION

Gastrointestinal stromal tumors (GIST) are the most frequent sarcoma of the gastrointestinal tract and arise from interstitial cells of Cajal (ICC) or their precursors [1]. ICC coordinate gut motility as they mediate inputs from the enteric nervous system and elicit pacemaker activity throughout the smooth muscle layers of the digestive tract [2, 3]. Smooth muscle cells (SMC) and ICC share common mesenchymal precursors expressing, the tyrosine kinase receptor KIT (c-kit, CD117), which remain expressed in ICC throughout life and is essential for their development and maintenance [4].

A somatic gain-of function mutation of KIT is present in approximately 85% of GIST [5]. The tyrosine kinase inhibitor (TKI) imatinib mesylate (STI571, Gleevec®) epitomizes the concept of targeted therapy in GIST but, despite encouraging initial results and ongoing development of novel TKIs, resistance and relapses remain the rule [6, 7], e.g. imatinib induces a form of quiescence in many imatinib-sensitive cells, eventually resulting in acquired resistance and treatment failure [8]. Progress in targeted therapy is bound to a better understanding of the underlying cellular and molecular mechanisms [9, 10]. Therefore, new targets and original therapeutic strategies are very much needed to improve outcome and treatment tolerability [11].

Both ICC and GIST express phosphodiesterase 3A (PDE3A) [12], a member of the large family of cyclic nucleotide phosphodiesterases (PDE) [13]. PDE hydrolyze cyclic adenosine monophosphate (cAMP) and cyclic guanosine phosphate (cGMP), thereby regulating their intracellular concentrations and, consequently, influencing myriads of biological responses in health and disease. PDEs expression and functions have been studied mainly in brain, heart, vascular SMC, platelets and oocyte [14]. Selective inhibitors of several PDE are already in clinical use for treatment of e.g. erectile dysfunction, pulmonary hypertension, cardiac failure, intermittent claudication or chronic obstructive pulmonary disease. Many new PDE inhibitors are being developed [15].

Recently, PDE3A has been brought into the spotlight in oncology. de Waal *et al.* identified cancer-cytotoxic modulators of PDE3A by predictive chemogenomics and demonstrated in HeLa cells that the strong viability reduction observed in cancer cell lines with the non-catalytic inhibitor DNMDP, was induced by an original neomorphic interaction between PDE3A and the protein Schlafen 12 (SLFN12) [16]. Nazir *et al.* showed high levels of PDE3A expression in different cancer cell lines such as colon carcinoma or lung adenocarcinoma and underlined higher sensitivity to PDE3 inhibitors leading to reduced cell viability compared to other cells lines expressing less PDE3A [17].

We have previously unraveled the original role of PDE3A in ICC development and in GIST physiopathology. In the mouse gut, PDE3A was expressed in the ICC/SMC mesenchymal precursors and in mature ICC along the gut and PDE3A loss-of-function (PDE3A^-/-^) led to a marked reduction of the ICC network. PDE3A immunoreactivity was detected in 92% of human GIST samples. In the imatinib-sensitive GIST882 cell line, the PDE3 inhibitor cilostazol (Pletal™), already in clinical use for cardiovascular indications, halved cell viability and, most interestingly, can do so in synergy with imatinib [12]. However, imatinib-resistant GIST cell lines had not been studied, nor compared to imatinib-sensitive cells. Moreover, the molecular mechanisms involved in PDE3A acting on GIST viability remained to be determined.

In this study, we firstly evaluated the importance of PDE3A function in the imatinib-resistant GIST48 cell line [18] using a catalytic and a non-catalytic PDE3 inhibitor, cilostazol [19] and DNDMP [16], respectively. Next, as GIST derive from ICC or their precursors, we investigated the phenotype of GIST882 and GIST48 cell lines and compared the expression of key differentiation markers and transcription factors after short- and long-term treatment with PDE3 and KIT inhibitors. Finally, we asked whether the YAP pathway could be involved in GIST proliferation. The role of the Hippo/YAP pathway is well-known in cell proliferation and differentiation as a point of convergence for several major signaling pathways such as Wnt, TGFβ or Notch [20]. YAP expression is regulated by the transcription Limb Expression 1 (LIX1) which also controls the differentiation of stomach mesenchymal precursors into SMC [21]. Moreover, numerous roles of YAP in various cancers have been described [22], especially in sarcoma [23], and targeting YAP, with inhibitors such as verteporfin [24] overcomes drug resistance in colon and pancreatic cancer cell lines [25, 26].

## RESULTS

### The combination of imatinib and cilostazol decreased viability of the imatinib-resistant GIST48 cell line, independently of cAMP

**Figure 1:**
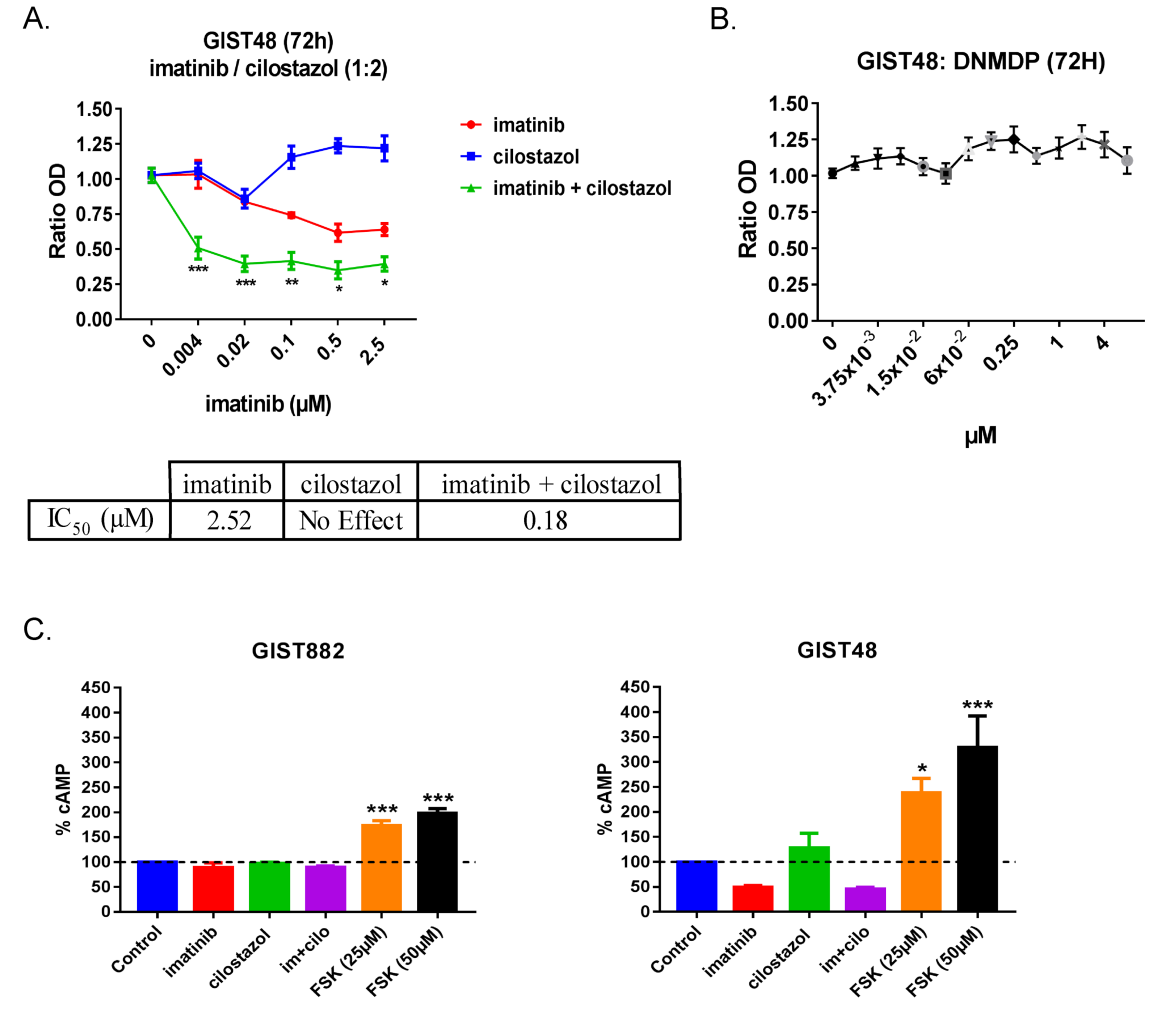
Cilostazol, a PDE3 inhibitor synergized with imatinib to reduce GIST48 cells viability **A)** WST-1 viability assay. Upper panel: GIST48 were treated with imatinib (0 to 2.5μM), cilostazol (0 to 5μM) and combination of the two at a 1:2 ratio (i.e. imatinib 0.5μM + cilostazol 1μM) for 72h. p-values (2-way ANOVA and Tukey’s post-hoc test). *: p ≤ 0.05, **: p ≤ 0.002, ***: p ≤ 0.001. Lower panel: IC50 for imatinib, cilostazol and combination of the two drugs showed the potentiation of imatinib effect by cilostazol in GIST48 cells. **B)** WST-1 viability assay. GIST48 were treated with a range of DNMDP concentrations for 72h. DNMDP did not affect GIST48 viability at any concentration tested. Mean values ± SEM from three independent experiments. **C)** cAMP accumulation in GIST882 (left panel) and GIST48 cells (right panel) treated for 72h with imatinib (1μM), cilostazol (10μM), imatinib + cilostazol (1μM + 10μM) and forskolin (25μM or 50μM). Imatinib, cilostazol or combination of the two drugs did not significantly affect cAMP levels in GIST882 and GIST48 cells. Forskolin, an adenylate cyclase activator used as positive control, significantly increased cAMP levels in both cell lines. All data presented as mean value ± SEM from four independent experiments. p-values (Kruskal-Wallis followed by Dunn’s test). **: p ≤ 0.002, ***: p ≤ 0.001.

To assess the effect of the PDE3 inhibitor cilostazol on cell viability in imatinib-resistant GIST cells, we treated imatinib-resistant GIST48 cells with a range of concentration of cilostazol or imatinib alone and a combination of cilostazol with imatinib (ratio 2:1) for 72h (Figure 1A). Imatinib showed an IC_50_ of 2.5μM, comparable with previously published data [27] (IC_50_ >1μM), while cilostazol alone did not affect GIST48 viability in the 0 to 25 μM range (Figure 1A).

Cilostazol potentiated the effect of imatinib on GIST48 cells viability reduction, as reflected by a particularly low IC_50_ of 0.18μM (Figure 1B).

In contrast with GIST882 cells, in which DNMDP exhibited an IC_50_ of 0.027μM [12], no reduction of viability was detected in GIST48 cells for DNMDP concentrations up to 4μM (Figure 1B).

As the best-characterized function of PDE3 is to hydrolyze cyclic nucleotides [15], we quantified the cAMP amount in GIST882 and GIST48 cells treated for 72h with 1μM imatinib, 10μM cilostazol and the combination of the two drugs (1μM imatinib +10μM cilostazol). No significant change in cAMP was observed in any condition, in contrast with the positive control, forskolin (Figure 1C), suggesting that cilostazol reduced GIST882 and GIST48 cell viability by a cAMP-independent mechanism.

### Low expression of PDE3A and SFPQ correlated with a myoid phenotype in imatinib-resistant GIST48 cell line, as compared with the imatinib-sensitive GIST882

Recently, Nazir *et al.* [17] unraveled the correlation between high PDE3A expression level and sensibility to PDE3 inhibitors in various solid cancers. To understand the absence of effect of cilostazol or DNMDP on GIST48 cells viability, expression level of PDE3A, its transcription factor SFPQ [28], PDE3B and SLFN12 were compared between GIST48 and GIST882 cells.

**Figure 2:**
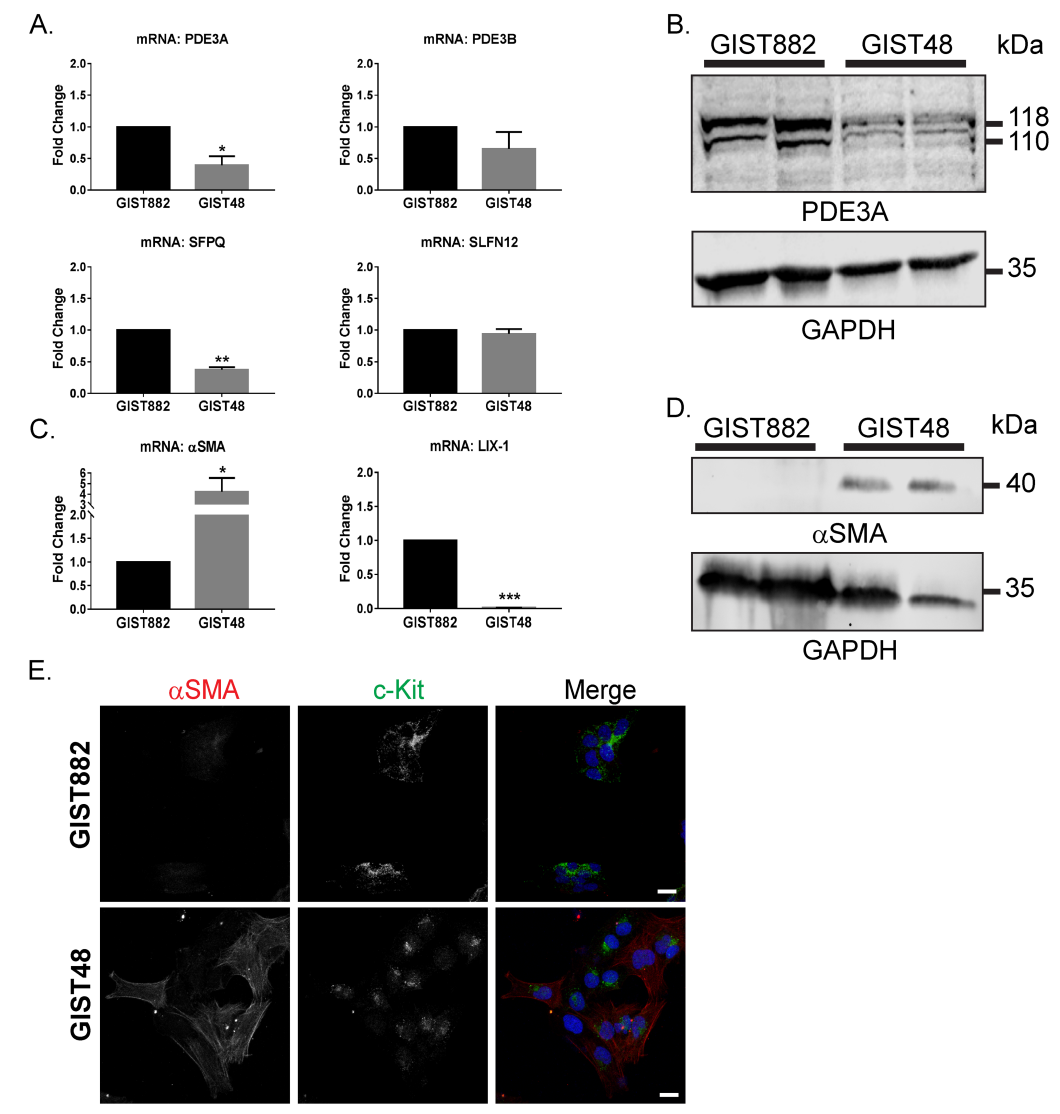
Differential expression of transcription factors and differentiation markers in GIST882 and GIST48 cells **A)** qPCR: mRNA level of PDE3A and its transcription factor SFPQ were significantly lower in GIST48 cells compared to GIST882 cells. PDE3B and SLFN12 expression were not statistically different. **B)** Western blot probed with anti-PDE3A and anti-GAPDH antibodies. Reduced PDE3A protein level in GIST48 cells as compared to GIST882 cells was observed. 50µg protein/lane. **C)** qPCR. αSMA mRNA level was significantly higher (fold change of 4) in GIST48 cells. Conversely, LIX-1 mRNA expression was lower in GIST48 cells compared to GIST882 cells. **D)** Western blot probed with anti-αSMA and anti-GAPDH antibodies. αSMA protein was undetectable in GIST882 cells but abundant in GIST48 cells. 50μg protein/lane. **E)** Immunofluorescence. Left panel: αSMA-ir. Middle panel: KIT-ir. Right panel: Merged image of αSMA-ir in red, KIT-ir in green and DAPI nuclear counterstain in blue. Both cells expressed KIT while more cells were αSMA positive in GIST48 (70-80%) compared to GIST882 (1-2%). Confocal microscopy. Scale bar = 20μm. qPCR data were obtained from five independent experiments and presented as mean value ± SEM. p-values (ratio paired t-test). *: p ≤ 0.05, **: p ≤ 0.002, ***: p ≤ 0.001. Western blot data and immunofluorescence data are representative for at least three independent experiments.

*PDE3A*, but not *PDE3B*, mRNA level was lower in GIST48 compared to GIST882 cells (Figure 2A). The lower PDE3A expression in GIST48 determined by qPCR was confirmed by Western blotting (Figure 2B). *SFPQ*, a transcription factor of PDE3A, was also less abundant in GIST48 cells. Conversely, for *SLFN12*, mRNA levels were similar between GIST48 and GIST882 cells (Figure 2A).

As GIST derive from either ICC or mesenchymal precursors which give rise to smooth muscle cells or ICC [5], we also compared the expression level of the myoid differentiation markers *LIX1*, a transcription factor expressed in mesenchymal progenitors [21], and (alpha) α-smooth muscle actin (αSMA), a smooth muscle cell marker.

GIST48 cells showed a high expression of αSMA mRNA and protein compared to GIST882 cells (Figure 2C and 2D). In line with these results, *LIX1* mRNA expression was barely detectable in GIST48 cells (Figure 2C). To further support these observations, we performed a double immunofluorescence staining for αSMA and KIT on both cell lines. KIT immunoreactivity was detected at the membrane and diffusely in the cytoplasm of GIST882 cells while in GIST48, KIT-ir was located in small clusters adjacent to the nucleus. Only 1-2% of GIST882 cells were positive for αSMA-ir, compared to 70-80% of αSMA-ir GIST48 cells, in line with the qPCR and Western blot data. Supplementary Table 6 summarizes gene expression and drug sensitivity of GIST882 and GIST48 cells.

### Nuclear location of YAP immunoreactivity is frequently shown in primary human GIST

**Figure 3:**
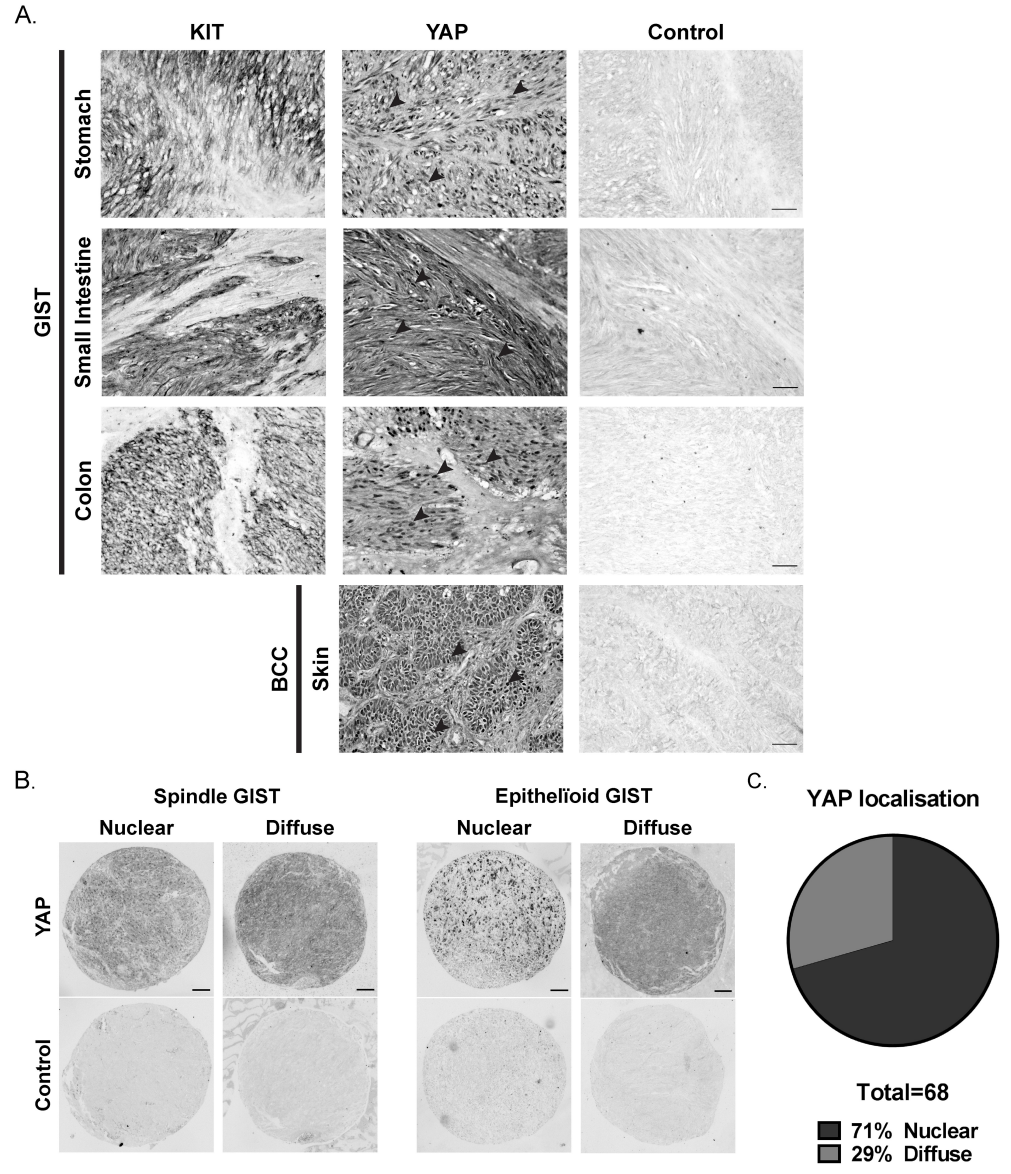
YAP expression in human GIST primary tumors **A)** Immunohistochemistry for KIT-ir and YAP-ir in three different human GIST primary tumors of stomach, small intestine and colon, respectively. Black arrowheads indicate nuclear staining of YAP-ir. Basal cell carcinoma (BCC) sample served as positive control for nuclear YAP location [30] (see Supplemental Table 5 for details). Scale bar = 50µm **B)** YAP immunohistochemistry on GIST tissue arrays (see Supplemental Table 4 for details). Representative examples of YAP-ir in spindle-shape and epithelioid GIST. scale bar = 100µm. **C)** Proportion of GIST samples exhibiting nuclear (48 cases) or diffuse (20 cases) YAP-ir.

McKey *et al.* showed that LIX1 regulates the expression and activity of YAP in stomach mesenchymal precursors [21]. Moreover, YAP appears to be important in sarcomas [23] and YAP controls PDE3A expression in liver cancer cells [29]. We therefore investigated YAP expression in human GIST tumors. Nuclear YAP-ir was observed in the KIT-positive region of five primary GIST samples as well as in basal cell carcinoma, used as positive control for nuclear YAP localization [30] (Figure 3A). In a GIST tissue microarray (TMA), 68 out of 75 samples (90%) were positive for YAP-ir, of which 48 (71%) showed a nuclear localization (Figure 3B), while 20 (29%) harbored a diffuse YAP-ir signal. (Figure 3C).

### Cilostazol, but not imatinib, leads to YAP nuclear exclusion in imatinib-sensitive GIST882 and imatinib-resistant GIST48 cells

**Figure 4:**
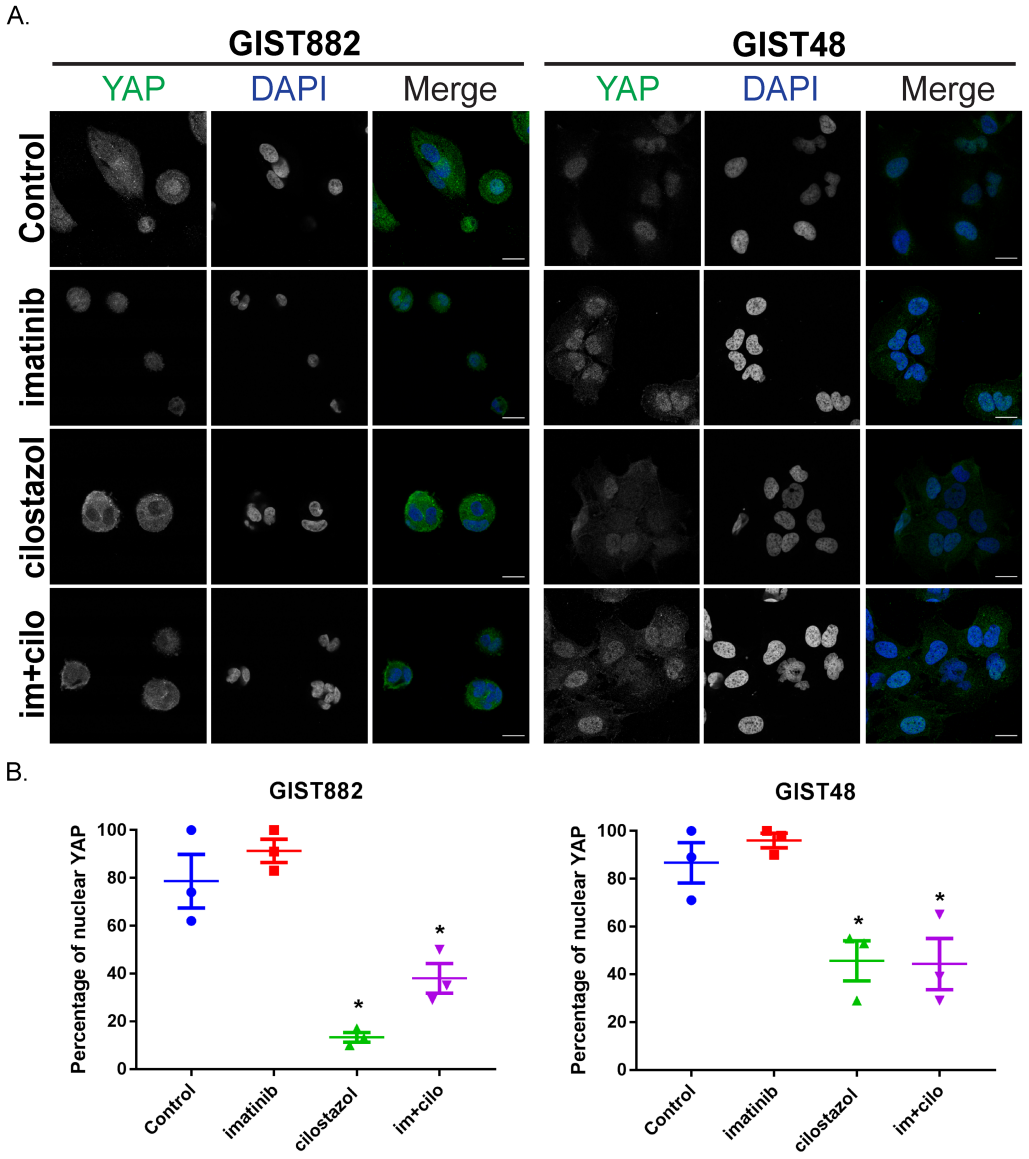
Cilostazol, but not imatinib, leads to YAP nuclear exclusion in imatinib-sensitive GIST882 and imatinib-resistant GIST48 cells GIST882 and GIST48 cells were treated without or with imatinib (1μM), cilostazol (10μM) or cilostazol + imatinib (10μM:1μM) for 72h. **A)** Immunofluorescence. Left panel: YAP-ir (in green) in GIST882 with DAPI nuclear counterstaining. Right panel: YAP-ir (in green) in GIST48 with DAPI nuclear counterstaining. Confocal microscopy. Scale bars = 20μm. **B)** Left panel: percentage of GIST882 cells with nuclear YAP-ir in. Right panel: Percentage of GIST48 cells showing nuclear YAP-ir. Treatment with cilostazol or imatinib + cilostazol reduced nuclear YAP-ir in both cell lines. Images are representative for three independent experiments and presented as mean value ± SEM. p-values (one-way ANOVA). *: p≤ 0.05.

To test if KIT or PDE3A inhibition influence YAP activity, we assessed YAP localization by immunofluorescence in GIST882 and GIST48 cells treated with imatinib, cilostazol and combination of the two drugs for 72h (Figure 4A). In GIST882 cells, cilostazol and the combination of imatinib and cilostazol reduced the nuclear location of YAP-ir compared to control or cells treated with imatinib alone (Figure 4B, left panel). In GIST48, nuclear location of YAP-ir- was also reduced by cilostazol or the combination of imatinib and cilostazol, compared to imatinib and untreated cells (Figure 4B, right panel).

### Verteporfin, a YAP inhibitor, decreased GIST882 and GIST48 cells viability by 90%

**Figure 5:**
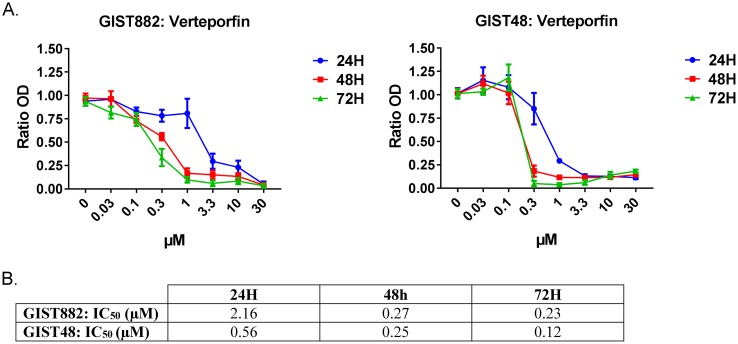
Verteporfin, a YAP inhibitor, reduced GIST882 and GIST48 cells viability **A)** WST-1 viability assay. GIST882 and GIST48 were treated with verteporfin (0 to 30μM) for 24h, 48h and 72h. **B)** The respective IC50 at each time-point for both cell lines. GIST882 and GIST48 cells were both sensitive to verteporfin, with a higher sensitivity for the imatinib-resistant GIST48 cells. Mean values from three independent experiments. Data presented as means ± SEM.

To assess the role of YAP pathway in GIST cells lines, we used verteporfin, an inhibitor of YAP/TEAD interaction [24]. Viability of GIST882 and GIST48 cells was measured after 24H, 48H and 72H of treatment with a range of verteporfin concentration from 0 to 30μM (Figure 5A). Both GIST882 and GIST48 cell lines showed high sensitivity to verteporfin. Noteworthy, the imatinib-resistant GIST48 cell line appeared consistently more sensitive to YAP inhibition than the imatinib-sensitive GIST882 cells, with IC_50_ at 24H (0.56 μM) already 4 times lower than IC_50_ for GIST882 (2.16 μM) (Figure 5B).

## DISCUSSION

In a previous study [12], we unraveled the novel role of PDE3A in ICC development and its involvement in GIST physiopathology. Important questions, such as the effect of cilostazol and DNMDP on imatinib-resistant GIST cells and the signaling pathways involved in the reduction of cell viability remained however unanswered.

### Cilostazol potentiated the effect of imatinib in imatinib-resistant GIST48 cells

Here, we provide original evidence that cilostazol has no noticeable effect on its own in the imatinib-resistant GIST48 cells, but potentiated the effect of imatinib, reducing cell viability by 50% in the nanomolar range of concentrations. Although that reduction of cell viability was incomplete, the adjunction of cilostazol lead to a remarkable tenfold reduction of the imatinib IC_50_ in the imatinib-resistant GIST48 cell line (Figure 1A), namely, IC_50_ for imatinib alone: ∼2.52 μM vs IC_50_ for imatinib + cilostazol: ∼0.18μM - in the range of imatinib IC_50_ in the imatinib-sensitive GIST882 cells (∼0.21 μM) [31].

As cilostazol competes with cAMP at the PDE3 catalytic site [32], acting thus as a competitive inhibitor of phosphodiesterase activity, we measured cAMP levels in GIST882 and GIST48 cells. No change of cAMP levels was observed after treatment, suggesting that, in our model, cilostazol acts in a non-catalytic way to reduce GIST cell viability. In line with our observation, cAMP-independent effects of cilostazol have been previously reported in, e.g., macrophages [33] or microglial cells [34], and other compounds, epitomized by DNMDP, also exert an allosteric, non-catalytic, regulation of PDE3 [16].

### Imatinib-resistant GIST48 cells have low PDE3A expression and a myoid phenotype compared to imatinib-sensitive GIST882 cells

In contrast with GIST882 cells, cilostazol or DNMDP did not affect GIST48 cells viability. We hypothesized that this lack of sensitivity might be linked to the lower PDE3A expression in GIST48 cells. A recent study from Nazir *et al.* [17] demonstrated in various solid cancer cell lines that PDE3A expression level is critical for sensitivity to PDE3 inhibitors. We found that both PDE3A mRNA and protein level were lower in GIST48 cells as compared to GIST882 cells, while SFPQ, a known transcription factor for PDE3A [28], was also repressed in GIST48 cells, in line with the lower PDE3A level in these cells. Those observations led us to further investigate their phenotype.

GIST derive from ICC or their mesenchymal precursors and we previously reported in the mouse embryo a timely and spatially regulated PDE3A expression during differentiation of the mesenchymal ICC/SMC precursors, with expression of PDE3A persisting in mature ICC but not in differentiated SMC [12]. Thus, we assessed in GIST882 and GIST48 cells the expression of the two main differentiation markers: KIT, expressed in mesenchymal precursors and in ICC, and αSMA, an early marker of smooth muscle cells [35]. As expected, both cell lines expressed KIT. In GIST48 cells KIT-ir was observed prominently in small clusters adjacent to the nucleus, a peculiar location previously reported in other resistant GIST cell lines and linked to KIT oncogenic mutations in the tyrosine kinase II domain, leading to KIT activation in the Golgi [36, 37]. αSMA-ir was observed in most GIST48 cells, while very few GIST882 cells were immunoreactive for αSMA. Absence of LIX1 expression, a transcription factor responsible of mesenchymal phenotype maintenance [21] together with low PDE3A expression, was in line with a myoid phenotype of GIST48 cells.

GIST882 cells present a phenotype close to the ICC phenotype (KIT+, PDE3A+, αSMA-) while the GIST48 cells phenotype may be closer to the phenotype of ICC/SMC mesenchymal precursors (Kit+, PDE3A low, αSMA+). GIST 48 cells [18] originate from a patient showing initial response under imatinib therapy but subsequently progressed under treatment. They harbor a homozygous KIT exon 11 mutation (V560D) and a heterozygous, secondary, KIT exon 17 mutation (D820A). The myoid phenotype of GIST48 cells echoes the presence of small cluster of cells expressing myoid markers in a subset of imatinib-responsive GIST tissues reported by Agaram *et al.* [38]. These clusters could be linked to the occurrence of relapse or progression after imatinib treatment.

Further experiments of loss or gain of function of PDE3A will be required to characterize the role of PDE3A in the phenotype and sensitivity to compounds targeting PDE3A in GIST cells.

### YAP, a major player in GIST cells viability

The link between expression of genes of mesenchymal ICC/SMC precursors and phenotype of GIST cell lines prompted us to consider the YAP pathway, involved in regulation of cells differentiation, proliferation or tissue growth [20]. YAP has been reported to be expressed in 50% of sarcomas [23] and YAP interaction with TFCP2 controls PDE3A expression [29]. In addition, LIX1 has been shown to control expression and activity of YAP [21]. YAP is an important transcriptional co-activator and its activity is tightly regulated by multiple upstream pathways. In the nucleus, YAP activates a complex transcriptional program in a cell type-dependent manner. Activation of the Hippo pathway leads to YAP phosphorylation and sequestration in the cytoplasm, hence YAP inactivation (“Hippo ON / YAP OFF”) [20, 39]. A nuclear location of YAP, indicating YAP activity, was observed in a majority (71%) of GIST primary tumors and in both imatinib-sensitive GIST882 and imatinib-resistant GIST48 cell lines.

Verteporfin, a YAP/TEAD inhibitor, lead to massive cell death in both GIST882 and GIST48 cell lines, suggesting that GIST 882 & GIST48 cells are “addicted” to YAP as the shutdown of YAP transcriptional activity overcomes the constitutive activation of survival pathways driven by KIT oncogenic mutations.

In both GIST882 and GIST48 cells, the PDE3 inhibitor cilostazol induced nuclear exclusion of YAP, indicative for the downregulation of YAP pathway activity. Cilostazol on its own reduced viability in GIST882 [12] cells, but not in GIST48 cells, while cilostazol remarkably enhanced the effect of imatinib in both cell lines.

A study by Yu *et al.* [40] suggested the use of PDE inhibitors to indirectly inhibit YAP pathway by activation of the Hippo pathway in a cAMP-PKA -dependent manner in the MDA-MB-231 human breast adenocarcinoma cell line. In contrast, we observed in GIST cell lines, that cilostazol effect on YAP inactivation/nuclear exclusion and reduction of cell viability was not accompanied by modification of cAMP levels, suggesting other, cAMP-independent, mechanism(s), such as allosteric inhibition already reported for PDE4 [41, 42] and for PDE3A by De Waal in HeLa cells [16].

To our best knowledge, downregulation of YAP by cilostazol has not been previously reported. The precise effectors of cilostazol action on YAP inactivation remain to be identified, e.g. by co-immunoprecipitation of PDE3A in presence of cilostazol to identify PDE3A interactors linked to the Hippo pathway upstream of YAP.

Verteporfin has a more drastic effect than cilostazol on GIST cell viability reduction. YAP is at the crossroads of multiple, interacting, upstream pathways, with multiple possible compensatory mechanisms, while verteporfin blocks directly, hence more efficiently, YAP activity at the level of the YAP/TEAD transcriptional complex.

Noteworthy, inhibition of oncogenic KIT pathways by imatinib did not affect the nuclear location of YAP-ir, suggesting that viability reduction elicited by imatinib is not mediated through down-regulation of the YAP pathway.

**Figure 6:**
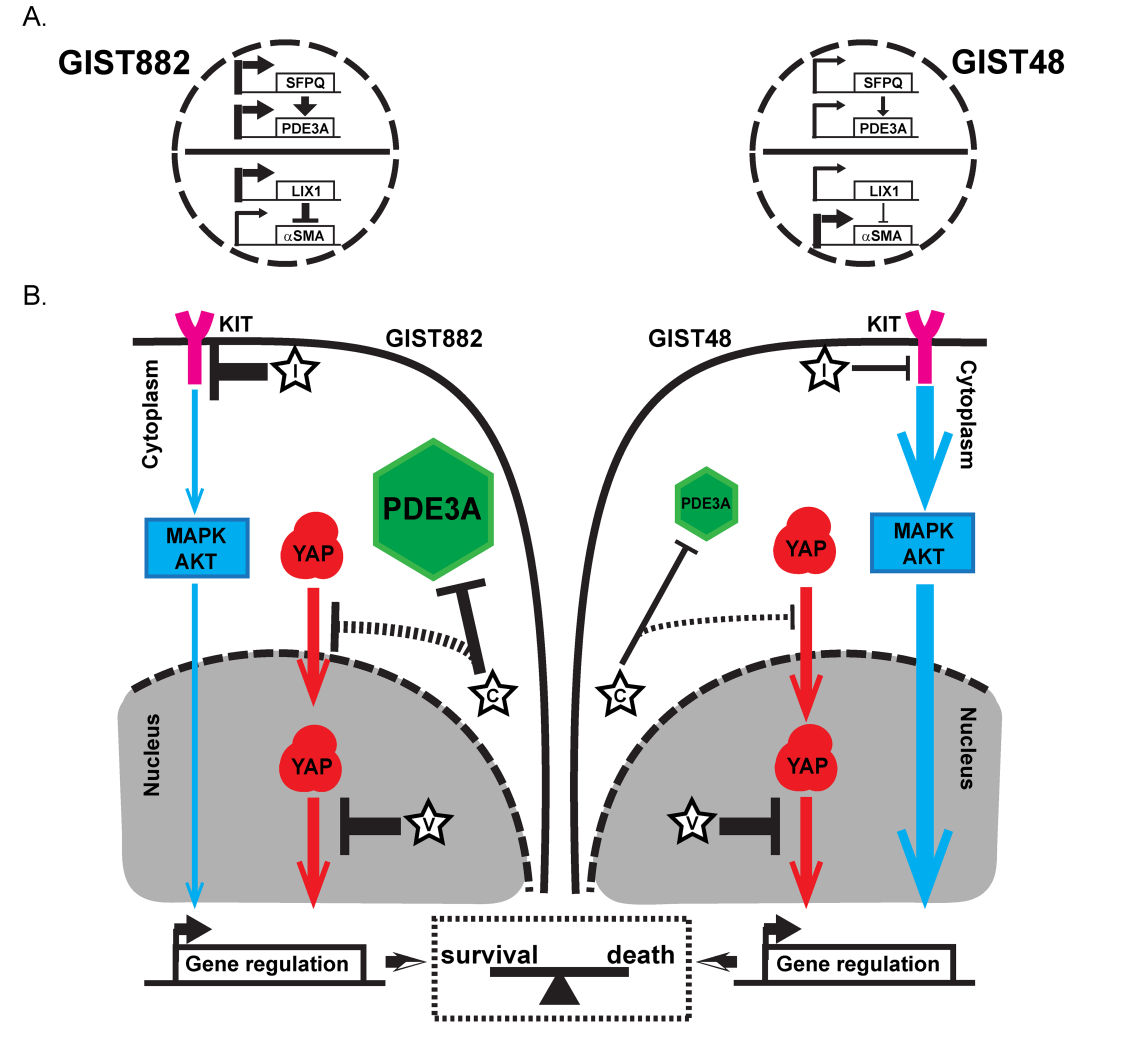
Regulation of differentiation genes expression and a model for dual survival pathways and addiction to YAP in GIST882 and GIST48 cells **A)** Regulation of expression of differentiation genes in GIST882 and GIST48 cells. Thick arrows indicate high gene expression of LIX1, SFPQ and PDE3A in GIST882 cells compared to GIST48 cells. High LIX1 expression downregulated expression of αSMA in GIST882, while low LIX1 expression led to higher αSMA expression in GIST48 cells. **B)** Model for dual survival pathways and addiction to YAP in GIST882 and GIST48 cells. C: cilostazol; D: DNMDP; I: imatinib; V: verteporfin. Plain black arrows: Effect of the different drugs on their targets (flat head = inhibition; arrow head= induction). Thick arrows indicate stronger effect of the drug. Dashed arrow indicates hypothetic effect of the drug. Blue arrows: KIT inhibition by imatinib led to downregulation of major pathways such as MAPK/ERK or PKB controlling expression of viability factors. Thick arrow indicates a stronger KIT oncogenic drive. Red arrows: verteporfin, a direct inhibitor of YAP/TEAD interaction shuts down YAP transcriptional program, causing severe cytotoxicity in both in GIST882 and GIST48 cells. In GIST882 cells, activation of oncogenic KIT downstream pathways is modest (i.e. sensitive to imatinib). Cilostazol leads to nuclear exclusion of YAP and reduces of cell viability, alone or in synergism with imatinib. In GIST48 cells, lower PDE3A expression and stronger activation of KIT downstream pathways (i.e. resistance to imatinib) overcomes the effect of cilostazol on YAP-driven viability. Cilostazol however sensitizes GIST48 cells to the modest inhibition of oncogenic KIT downstream pathways by imatinib. Our data suggest that oncogenic KIT downstream pathways and YAP pathway act in parallel to drive cell survival. In case of simultaneous inhibition of the two pathways, their relative strength shifts the balance between survival and cell death.

A model emerges (cartoon Figure 6) in which GIST cells depend for their survival/proliferation on two pathways, KIT-MAPK/ERK and YAP, in parallel, the outcome (death/survival) being a matter of balance and relative strength of the two drivers. GIST882 cells, with a modest KIT oncogenic activation (“sensitive” to inhibition by imatinib) and a sustained YAP activation, are sensitive to imatinib (efficient KIT inhibition) and to cilostazol (indirect, modest, YAP inhibition) with synergism of imatinib and cilostazol on viability reduction. GIST48 cells, with a strong KIT oncogenic activation (“resistant” to inhibition by imatinib) and sustained YAP activation, are resistant to imatinib (IC_50_ >10 fold higher compared to GIST882 cells) and to cilostazol (indirect, modest, YAP inhibition) while simultaneous inhibition of both pathways by imatinib + cilostazol elicits potentiation and viability reduction.

Assessing other GIST cell lines carrying different oncogenic mutations *in vitro* and mouse xenograft models *in vivo* will shed additional light on the molecular mechanisms involved, allowing to refine the proposed model in the future.

### Translational perspective

Drug repurposing [43], a.k.a. drug repositioning, has been vividly described as "*Inventing new therapies without reinventing the wheel*" [44]. The development of a new compound for clinical use is a lengthy, costly process with a very low success rate. To address, on a shorter time-span, the patient’s needs for better therapy, drug repurposing, which aims to use existing drugs to treat conditions for which they were not originally intended, has the potential to provide a faster, cheaper and more certain route to clinical approval [44, 45]. Multiple examples of drug repurposing exist in various domains, including cancer therapy [45] and even in GIST [46].

Cilostazol is already approved by the FDA for clinical use in indications such as intermittent claudication or platelet aggregation [47].

Nowadays, imatinib resistance in GIST is usually addressed with escalation of the imatinib dose (i.e. 800 mg daily instead of 400 mg daily), leading to a significant increase of adverse effects, with a negative impact on quality of life and compliance to treatment [2]. Achieving a response with a lower imatinib dose by adjunction of cilostazol could be of great clinical interest in the context of resistant tumors.

*In vitro* results on cell lines or mouse xenograft models *in vivo* do not readily translate into clinical response *in vivo*. Based on our original observations in GIST48 cells, a phase I clinical trial (« *cilostazol as imatinib synergiser in patients with unresectable or metastatic GIST treated by Glivec*® » - CILOGIST - EudraCT: 2018-001295-37), has been recently (October 2018) initiated at the Erasme Academic Hospital, Université Libre de Bruxelles, Belgium (Prof. Jean-Luc Van Laethem & Dr. Anne Demols, Clinique d’Oncologie Digestive, Service de Gastroentérologie, Prof. Jacques Devière).

The YAP/TEAD inhibitor verteporfin also emerges as a new valuable prospect for GIST therapy. Verteporfin [24] enhances imatinib effect in leukemia [48] and overcomes *in vitro* resistance to various drugs in other cancer cells as well [25, 26]. Clinical trials with verteporfin or other YAP inhibitors might thus be considered for GIST in the future.

In conclusion, our original observations that cilostazol potentiates imatinib viability reduction not only in the imatinib-sensitive GIST882 cell line but also in the imatinib-resistant GIST48 cell line on one hand, and that YAP is a major driver in GIST cell lines and in primary GIST on the other, open exciting translational perspective for drug repurposing in GIST targeted therapy.

## MATERIALS AND METHODS

### Ethics statement

The collection and analysis of human tissue samples has been conducted in accordance with the ethical standards and according to the Declaration of Helsinki and according to national and international guidelines. The study has been approved (DACE P2016-316) by the Institutional Medical Ethics Committee of Erasmus Hospital and Faculty of Medicine, Université Libre de Bruxelles, Brussels, Belgium.

### Cell lines and drugs

The human GIST882 cell line [49] was kindly provided by Dr. Jonathan A. Fletcher, Harvard Medical School, Boston, MA, USA. Cells were cultured at 37°C in DMEM (GIBCO, CA, USA) supplemented with 10% FBS, 2% penicillin/streptomycin. The human GIST48 cell line [18] was kindly provided by Dr. Ronald DeMatteo, Perelman School of Medicine, Philadelphia, PA, USA. Cells were cultured at 37°C in RPMI-1640 (GIBCO, CA, USA) supplemented with 10% FBS, 2 mM L-glutamine, 50 U/mL penicillin-streptomycin, 0.1% 2- mercaptoethanol, and 10 mM Hepes. Cilostazol, forskolin and verteporfin were purchased from Sigma, St. Louis, MO, USA, imatinib was purchased from LC Laboratories, Woburn, MA, USA, and DNMDP was purchased from Aobious Inc, Gloucester, MA, USA.

### Cell viability assay

Cell viability was assayed using a WST-1 assay (Roche, Indianapolis, IN, USA). GIST882 and GIST48 cells were seeded in 96-well plates (TPP Techno Plastic Products AG, Trasadingen, Switzerland) at a concentration of 10,000 cells/well supplemented with 100μl of medium 48h before drug treatments. After drug treatment, 10μl of WST-1 reagent was added and plates were incubated for 2h at 37°C. Absorbance was measured at 450 nm on a plate reader (iMark Microplate Absorbance Reader, BioRad, Hercules, CA, USA). IC_50_ were calculated thanks to Prism 7 software (GraphPad Software, Inc., La Jolla, CA, USA).

### cAMP measurement

GIST882 and GIST48 cells were seeded in 6-well plates (TPP Techno Plastic Products AG, Trasadingen, Switzerland) at a concentration of 250,000 cells/well, 48h before drug treatments. After drug treatments, cell medium was removed and 1ml of HCl 0.1M was added. cAMP was measured by RIA as described [50].

### Immunofluorescence (IF)

Cells were washed with 0.01M PBS pH 7.4 and fixed for 30 min in fresh 4% paraformaldehyde, pH 7.4 then washed two times with 0.01M TBS, pH7.4 and stored in 0.01M TBS/Sodium azide 0.1% solution until use.

For immunostaining, slides were brought to RT, permeabilized and blocked for 1h in 0.01M TBS pH 8.2 containing 0.1% Triton X-100 (Sigma, Saint Louis, MO, USA) and 10% normal horse serum (NHS). Primary antibodies were diluted in a TBS-Triton X-100 0.1% and 1% NHS solution and incubated overnight at RT in a humid chamber. Slides were washed in TBS and incubated at RT for 1 hour in TBS containing the secondary antibodies. Slides were washed and mounted using Glycergel (Dako, Glostrup, Denmark) + 2.5% DABCO (Sigma). For YAP quantification, the ratio between the cytoplasmic YAP mean fluorescence by the nuclear YAP mean fluorescence has been calculated. YAP localization was considered to be nuclear when the ratio was <1.

### Human GIST FFPE slides and tissue microarrays (TMA)

A cohort of GIST microarray slides of formalin-fixed, paraffin-embedded (FFPE) material were used. Slides, purchased from CMMI-DiaPath (Center for Microscopy and Molecular Imaging, Gosselies, Belgium), originates from the Department of Pathology, Erasmus Academic Hospital, Université Libre de Bruxelles, Brussels, Belgium. It contained a total of 75 FFPE human GIST tissue specimens including 8 metastatic specimens. Clinicopathological features are given in Supplementary Table 2. GIST primary tumor and BCC slide were obtained from the Laboratory of Pathological Anatomy, Jules Bordet Institute, Brussels, Belgium. Clinicopathological features are given in Supplementary Tables 4 and 5.

For immunohistochemistry, FFPE slides were rehydrated through phenol and graded alcohol solutions then heated at 96°C in 1 mM EDTA, 0.05% Tween20 (pH 8.0) antigen retrieval solution for 20 min to achieve epitope unmasking. Slides were then cooled for 10 min before being put in a 0.1% H_2_O_2_/Methanol solution for 30 min in order to block endogenous peroxidase. After washing, primary antibodies diluted in a TBS-Triton X-100 0.1% and 1% NHS solution were incubated overnight at RT in a humid chamber. Sections were rinsed and incubated with a secondary biotinylated antibody for 1h then with an ABC solution (ABC kit standard PK-4000; Vector Laboratories, Burlingame, CA, USA) for 1h. Revelation with nickel-enhanced DAB (DAB-Ni) was performed at room temperature for 5–10 min, resulting in a black precipitate. The DAB-Ni solution was prepared by dissolving 0.06 g of nickel ammonium sulphate (Fluka, Buchs, Switzerland) and 2 mg of DAB (Sigma-Aldrich) in 10 ml of 0.05 M Tris/HCl, pH 8. Immediately before use, 1 μl of 30% H_2_O_2_ (Merck, Darmstadt, Germany) was added. Tissue was evaluated by three examiners and immunoreactivity was considered as positive when signal was above signal in the negative control. List of antibodies used is given in Supplementary Table 1

### Confocal microscopy

High resolution imaging was performed using a Zeiss LSM780 system fitted on an Observer Z1 inverted microscope equipped with a LD LCI C-Apochromat 40x/1.1W objective (Zeiss). The 488 nm excitation wavelength of the Argon/2 laser, a main dichroic HFT 488 and a band-pass emission filter (BP490-535 nm) were used for selective detection of the green fluorochrome. The 543 nm excitation wavelength of the HeNe1 laser, a main dichroic HFT 488/543 and a long-pass emission filter (BP553-624 nm) were used for selective detection of the red fluorochrome. A 405 nm blue diode, a main dichroic HFT 405 and a band-pass emission filter (BP415-468 nm) were used for selective detection of the DNA counterstain. Single optical sections were acquired sequentially with a zoom factor of 1.5 and optimal (1 Airy unit) pinhole (scaling (x-y-z): 0.21 x 0.21 x 0.53 micron) and stored as 8-bit proprietary czi files. Single plane images were displayed using Zen2010 software (Zeiss) and exported as 8 bits uncompressed TIF images.

### Western blot

Cell samples were lysed for 2h at 4°C in lysis buffer containing 0.01M Tris-HCl (pH 7.4), 0.15M KCl, 0.1M NaF, 0.002M EDTA, 0.012M β-mercaptoethanol, 0.5% Nonidet P-40 and a cocktail of protease inhibitors (leupeptin 0.01 mg/ml; 0.001M Na_3_VO_4_; Pefabloc 0.3 mg/ml; 0.01μM okadaic acid). Proteins were denatured in sample buffer, heated at 95°C for 1 min, separated by SDS-PAGE on 10% polyacrylamide gel and transferred on a 0.2 μm nitrocellulose membrane. Primary antibodies raised in different species and secondary antibodies coupled with different fluorochromes, were sequentially combined to specifically label one marker in green (800 Li-Cor), the other in red (680 Li-Cor). The Azure c500 imaging system (Azure Biosystems, Dublin, CA, USA) was used to quantify the signals by immunofluorescence detection. List of antibodies used is given in Supplementary Table 2.

### Real time quantitative PCR (qPCR)

RNA samples from untreated GIST882 and GIST48 cells were used. Total RNA was extracted using RNeasy MiniKit (Qiagen, Valencia, CA, USA) according to the manufacturer’s instructions. Genomic DNA was removed using the RNase- Free DNase set (Qiagen). RNA was reverse transcribed with 200 units of M-MLV Reverse Transcriptase (Invitrogen, Eugene, Oregon, USA) in a reaction containing 1μg of random primers (Amersham Bioscience, Piscataway, NJ, USA), 0.01M each dNTP, 1x First-Strand buffer and 0.1M dithiothreitol followed by heat deactivation. The cDNA reverse transcription product was amplified with specific primers (Supplementary Table 2) by qPCR using SYBR Green chemistry on a 7500 Real-time PCR system (Applied Biosystems, Foster City, CA, USA). Identical thermal profile conditions, namely 95°C for 10min, then 40 cycles of 95°C for 15sec and 60°C for 1min were used for all primer sets. Emitted fluorescence was measured during annealing/ extension phase and amplification plots were generated using the Sequence Detection System. Transcriptional quantification relative to GAPDH and β-actin reference genes was performed using qBase+ software (Biogazelle, Zwijnaarde, Belgium). List of primers used is given in Supplementary Table 3.

### Statistics

Statistical analysis was performed with Prism 7 software (GraphPad Software, Inc., La Jolla, CA, USA), using Kruskal-Wallis followed by Dunn’s test for cAMP measurement, 2-way ANOVA and Tukey’s post-test for viability assay, Ratio paired t-test for qPCR and D'Agostino & Pearson normality test followed by ordinary One-way ANOVA for immunofluorescence quantification. P-values under 0.5 were considered as significant.

## SUPPLEMENTARY MATERIALS TABLES



## Abbreviations

αSMA: Alpha smooth muscle actin
cAMP: Cyclic adenosine monophosphate
GIST: Gastrointestinal stromal tumor
ICC: Interstitial cell of Cajal
LIX-1: Limb and CNS Expressed 1
PDE: phosphodiesterase
SFPQ: Splicing factor, proline- and glutamine-rich
SMC: Smooth muscle cell
TKI: Tyrosine kinase inhibitor
YAP: Yes-associated protein
