# Supplementary material for: Potentiation of imatinib by cilostazol in sensitive and resistant gastrointestinal stromal tumor cell lines involves YAP inhibition
Source: Oncotarget. 2019 Mar 5;10(19):1798–811. doi: 10.18632/oncotarget.26734 (PMC6442998; doi:10.18632/oncotarget.26734)
Supplement: Supplementary file 1 [file oncotarget-10-1798-s001.pdf]

Potentiation of imatinib by cilostazol in sensitive and resistant gastrointestinal stromal tumor cell lines involves YAP inhibition

SUPPLEMENTARY MATERIALS

Supplementary Table 1: Primary and secondary antibodies for immunostaining

Primary antibodies	Supplier	Cat. N°	Host	Dilution
PDE3A	MRC-PPU Reagents, Dundee, United Kingdom	S721A	Sheep	1/2000
αSMA	Sigma, St. Louis, MO, USA	C6198	Mouse	1/30.000
KIT (D13A2)	Cell signaling technology, Danvers, MA, USA	3074	Rabbit	1/500
KIT	DAKO, Glostrup, Denmark	A4502	Rabbit	1/500
YAP (1A12)	Cell signaling technology, Danvers, MA, USA	12395S	Mouse	1/500
**Secondary antibodies**	**Supplier**	**Cat.N°**	**Host**	**Dilution**
Anti-rabbit Biotin-SP	Jackson Immunoresearch laboratories, Inc.	711-065-152	Donkey	1/200
Anti-mouse Alexa 488	Jackson Immunoresearch laboratories, Inc.	715-545-151	Donkey	1/200
Anti-rabbit Alexa 488	Jackson Immunoresearch laboratories, Inc.	711-545-152	Donkey	1/200

Supplementary Table 2: Primary and secondary antibodies for Western blot

Primary antibodies	Supplier	Cat. N°	Host	Dilution
PDE3A	MRC-PPU Reagents	S721A	Sheep	1/500
αSMA	Sigma, St. Louis, MO, USA	C6198	Mouse	1/10.000
YAP (1A12)	Cell signaling technology	12395S	Mouse	1/500
GAPDH (14C10)	Cell signaling technology	2118	Rabbit	1/200
**Secondary antibodies**	**Supplier**	**Cat.N°**	**Host**	**Dilution**
Anti-sheep Alexa 680	Jackson Immunoresearch laboratories, Inc.	713-625-147	Donkey	1/5000
Anti-rabbit 680	Invitrogen, Watham, MA, USA	35568	Donkey	1/10.000
Anti-mouse 800	Invitrogen, Watham, MA, USA	SA535521	Donkey	1/10.000

Supplementary Table 3: Primers used for qPCR

Primers	Sequence
GAPDH Fw	ACCCACTCCTCCACCTTTGAC
GAPDH Rev	CATACCAGGAAATGAGCTTGACAA
B-Actin Fw	CATCCACGAAACTACCTTCAACTCC
B-Actin Rev	GAGCCGCCGATCCACAC
PDE3A Fw	TTTCCTTAGAGAGGTTCAAGGTCG
PDE3A Rev	AATACTGGTTCCTGAAGACTGTGAT
PDE3B Fwd	TACAATGCCCCTCAGGCAG
PDE3B Rev	GGCATACCAAGAGGCGATCA
SLFN12 Fw	GCAAGCCAACCAGGAGAAAAG
SLFN12 Rev	GGTAATCCGCAGACCAGAGG
SFPQ Fwd	GAGGAGAAGATCTCGGACTCG
SFPQ Rev	CGACATCGCTGTGTGTAAGTTT
LIX1 Fwd	GTGTTGGGGCCTATCACTAC
LIX1 Rev	GAGATGACTTCCTGTCGGG
αSMA Fw	TATCCCCGGGACTAAGACG
αSMA Rev	CACCATCACCCCCTGATGTC

Supplementary Table 4: Clinicopathologic features of CMMI DiaPath GIST TMA

**CMMI DiaPath GIST TMA.**	
**Sex/Age average**	**Total (n)/years**
Male	34/58.3
Female	22/62.6
Primary tumor site	**Total (n)**
Gastric	26
Small bowel	22
Colon	1
Disseminated	5
Tumor morphology	**Total (n)**
Spindle	64
Epithelioid	11
Risk category	**Total (n)**
Malignant	14
High risk	12
Intermediate risk	9
Low risk	21
Mitotic Figures	**Total (n)**
≤5/50	32
>5/50	24
KIT-ir	**Total (n)**
positive	67
negative	8

Supplementary Table 5: Jules Bordet Institute's FFPE GIST TMA slide: Clinicopathological features

Code	Age	Sex	Location	Morphology	Diagnostic	Type	Mitotic index
16B11851	84	M	Small Intestine	Spindle	Very low risk	Primary	$<5/5mm^2$
13B09403	70	F	Duodenum	Spindle	Low risk	Primary	No mitosis
18B13096	70	M	Left colon	Spindle	High risk	Primary	$>5/5mm^2$
18B10990	60	F	Stomach	Spindle	Low risk	Primary	$<5/5mm^2$
17B25663	67	F	Stomach	Spindle	High risk	Primary	$>5/5mm^2$

Supplementary Table 6: Summary of gene expression and drug response in GIST882 and GIST48 cell lines

Cell Line	Gene Expression					
	PDE3A	PDE3B	SLFN12	SFPQ	αSMA	LIX-1
GIST882	++	++	++	++	++	++
GIST48	+	++	++	+	+++	-

	Response to compounds			
	Imatinib	Cilostazol	Imatinib + Cilostazol	DNMDP
GIST882	+	+	+	+
GIST48	-	-	+	-
